# Analysis of cell–cell interaction between mural granulosa cells and cumulus granulosa cells during ovulation using single‐cell RNA sequencing data of mouse ovary

**DOI:** 10.1002/rmb2.12564

**Published:** 2024-02-14

**Authors:** Yuichiro Shirafuta, Isao Tamura, Amon Shiroshita, Taishi Fujimura, Ryo Maekawa, Toshiaki Taketani, Norihiro Sugino

**Affiliations:** ^1^ Department of Obstetrics and Gynecology Yamaguchi University Graduate School of Medicine Ube Japan

**Keywords:** cell interaction, cumulus cells, granulosa cells, ovary, single‐cell RNA‐seq

## Abstract

**Purpose:**

We investigated the interactions between mural granulosa cells (MGCs) and cumulus granulosa cells (CGCs) during ovulation after the LH surge.

**Methods:**

We performed clustering, pseudotime, and interactome analyses utilizing reported single‐cell RNA sequencing data of mouse ovary at 6 h after eCG‐hCG injection.

**Results:**

Clustering analysis classified granulosa cells into two distinct populations, MGCs and CGCs. Pseudotime analysis divided granulosa cells into before and after the LH surge, and further divided them into two branches, the ovulatory MGCs and the ovulatory CGCs. Interactome analysis was performed to identify the interactions between MGCs and CGCs. Twenty‐six interactions were acting from CGCs toward MGCs, involving ovulation and steroidogenesis. Thirty‐six interactions were acting from MGCs toward CGCs, involving hyaluronan synthesis. There were 25 bidirectional interactions, involving the EGFR pathway. In addition, we found three novel interactions: Ephrins–Ephs pathway and Wnt–Lrp6 pathway from CGCs to MGCs, associated with steroidogenesis and lipid transport, respectively, and TGF‐β–TGFBR1 pathway from MGCs to CGCs, associated with hyaluronan synthesis.

**Conclusions:**

MGCs and CGCs interact with each other in the preovulatory follicle after the LH surge, and their interactions have roles in corpus luteum formation, oocyte maturation, and follicle rupture.

## 1 INTRODUCTION

Ovarian granulosa cells differentiate into two distinct populations during follicular antrum formation, mural granulosa cells (MGCs), which line the follicular wall, and cumulus granulosa cells (CGCs), which surround the oocyte and form the cumulus–oocyte complex (COC). The two populations have different fates after the surge of luteinizing hormone (LH). After the LH surge, MGCs acquire endocrine functions such as steroidogenesis and contribute to the formation of the corpus luteum. At the same time, CGCs synthesize a mucoid intercellular matrix and undergo expansion, which contributes to oocyte maturation.^1^ It has been reported that these cells interact with each other, and their interactions are involved in the processes of corpus luteum formation, oocyte maturation, and follicle rupture.^2^, ^3^, ^4^ It is well known that epidermal growth factor (EGF)‐like factors produced by MGCs act on CGCs to promote oocyte maturation.^5^, ^6^, ^7^ However, detailed interactions between MGCs and CGCs are not fully understood. It is especially unclear how CGCs interact with MGCs.

Single‐cell RNA sequencing (scRNA‐seq) has been used in various tissues to understand gene expression and cellular diversity. In mouse ovaries, Park et al. reported the role of progesterone receptor in ovulation after the LH surge using an scRNA‐seq approach.^8^ Although there have been other reports of single‐cell RNA sequencing in mouse ovaries,^9^, ^10^, ^11^, ^12^ the changes in the interactions between MGCs and CGCs during ovulation remain unclear. In this study, we performed in silico analysis of the reported scRNA‐seq data of mouse ovary to investigate the interactions between MGCs and CGCs during ovulation after the LH surge.

## 2 MATERIALS AND METHODS

### 2.1 Isolation of MGCs and CGCs

This study was reviewed and approved by the Committee for Ethics on Animal Experiments at Yamaguchi University Graduate School of Medicine. All experiments were performed in accordance with relevant guidelines and regulations. C57BL/6 female mice (21‐ to 28‐day old) were purchased from Japan SLC (Shizuoka, Japan). They were injected intraperitoneally with 4 IU of equine chorionic gonadotropin (eCG) (Aska animal health, Tokyo, Japan) to promote follicular growth followed by 5 IU of human chorionic gonadotropin (hCG) (Aska animal health) injection to induce ovulation and luteinization. The ovaries were obtained before hCG (0) and 4, 8, and 12 h after hCG injection. The follicles were punctured in cold PBS, and MGCs and COCs were collected separately by pipette as reported previously.^13^ CGCs were stripped from oocytes by pipetting COCs in 0.1% Hyaluronidase (Sigma‐Aldrich, Bornem, Belgium). The MGCs and CGCs were centrifuged at 800 × *g*, pelleted, washed in cold PBS twice, and used for the real‐time RT‐PCR, respectively.

### 2.2 Real‐time RT‐PCR

Total RNA was isolated from granulosa cells with an RNeasy® Mini Kit. (QIAGEN, Valencia, CA). RT reaction and real‐time RT‐PCR were performed as reported previously.^14^, ^15^, ^16^ Gapdh was used as an internal control. Primer sequences are listed in Table S1.

### 2.3 Bioinformatics

To obtain a single‐cell transcriptome atlas and define the different cell types in the mouse ovary, we analyzed the single‐cell RNA sequencing data (GEO accession number: GSM4306338) reported by Park et al.^8^ In their study, 3‐week‐old immature mice were injected with eCG (5 IU) followed by hCG (5 IU). Six hours after hCG injection, eight ovaries from four different mice were subjected to single‐cell RNA sequencing.

Feature barcode matrices were imported using Read10x function and CreateSeuratObject function in Seurat (version 4.1.0).^17^ For each cell, a minimum expression of 500 genes was applied to filtered uninformative cells. For each gene, a minimum of five‐cell expression was applied. Gene expression levels were calculated as log‐transformed counts using NormalizeData. After normalization, dimensionality reduction and visualization for the 10x data were performed using Uniform Manifold Approximation and Projection (UMAP) for cells.^18^ The top 50 principal components of the variable genes were used as input of the UMAP algorithm with the default settings. Clustering on the UMAP embedding was performed using FindClusters function with “resolution = 0.5”. The marker genes were detected from each cluster using FindMarkers function. Ovarian cell types were determined by established cell type‐specific marker genes.

Pseudotime analysis for granulosa cells was performed using Monocle (version 2.22.0).^19^ For pseudotime analysis, the normalized data from the indicated clusters calculated by Seurat were directly imported into Monocle2. Granulosa cells were visualized by t‐SNE (num_dim = 25). Differentially expressed genes in each cluster ordered by *q*‐value and top 1000 genes were used for ordering cells. Dimensionality reduction was performed by reduceDimension function with DDRTree algorithm. Differential expression tests for each gene were performed by likelihood ratio tests, and each cell was assigned to branches by branchTest function. Gene expression levels in each branch were visualized by plot_genes_branched_heatmap function.

Interactome analysis was performed using CellChat (version 1.1.3)^20^ with default parameters. The normalized data from the indicated clusters calculated by Seurat were directly imported into CellChat. The strength of cell–cell interaction was calculated by “Communication Probability”. We added 0.01 to the Communication Probability value before the following calculation. The interactions whose Communication Probability increased 10‐fold compared to granulosa cells before LH surge were identified.

### 2.4 Statistical analysis

Tukey–Kramer test was applied to analyze the statistical differences between groups. Student *t*‐test was applied to analyze the difference between the two groups. All statistical analyses were performed using R (version 4.0.2, R Foundation for Statistical Computing, Vienna, Austria). Differences were considered significant at *p* < 0.05.

## 3 RESULTS

### 3.1 Identification of cell population of mouse ovary based on single‐cell gene expression profiles

**FIGURE 1:**
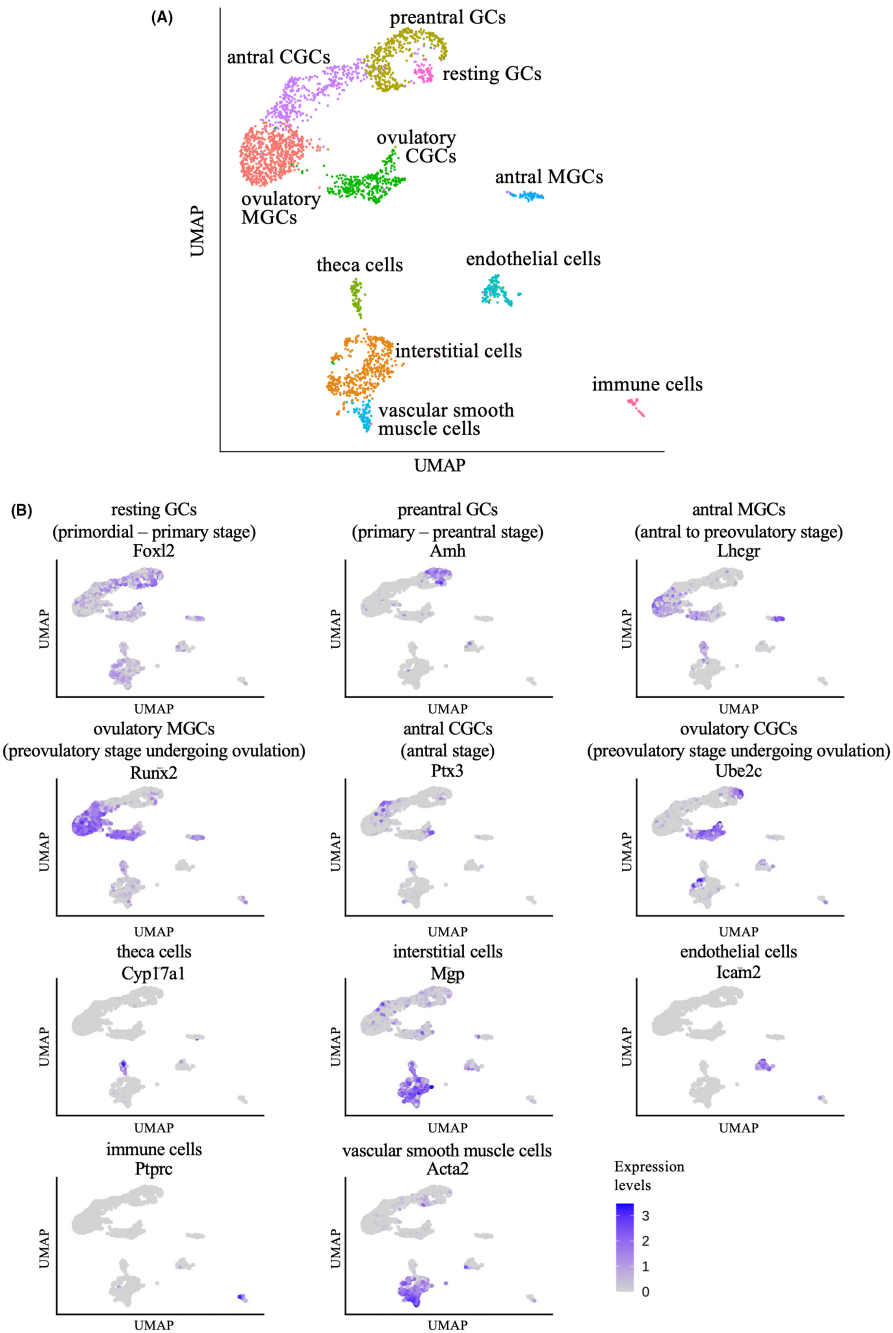
Identification of cell populations in mouse ovary based on single‐cell transcriptomes. (A) UAMP plot of all cells obtained from scRNA‐seq data of mouse ovary. Cells were colored according to clusters identified by Seurat clustering analysis and annotated based on marker genes which are characteristic of the indicated cell types. (B) UMAP plots showing the expression levels of marker genes of each cell type. Cells were colored according to the gene expression levels of each cell examined by Seurat.

Figure 1A shows a UMAP plot of single‐cell transcriptomes from mouse ovaries. A clustering analysis based on gene expression profiles identified 11 cell populations (Figure 1A). According to the expression of the established marker genes, each cell population was defined as follows: resting GCs from primordial to primary follicles (*Foxl2*),^21^ preantral GCs from primary to preantral follicles (*Amh*),^22^ antral MGCs from antral to preovulatory follicles (*Lhcgr*),^23^ ovulatory MGCs from preovulatory follicles undergoing ovulation (*Runx2*),^24^ antral CGCs from antral follicles (*Ptx3*),^25^ ovulatory CGCs from preovulatory follicles undergoing ovulation (*Ube2c*),^26^ theca cells (*Cyp17a1*),^27^ interstitial cells (*Mgp*),^28^ endothelial cells (*Icam2*),^29^ immune cells (*Ptprc*),^30^ and vascular smooth muscle cells (*Acta2*)^31^ (Figure 1B).

### 3.2 Differentiation of granulosa cell populations by pseudotime analysis

**FIGURE 2:**
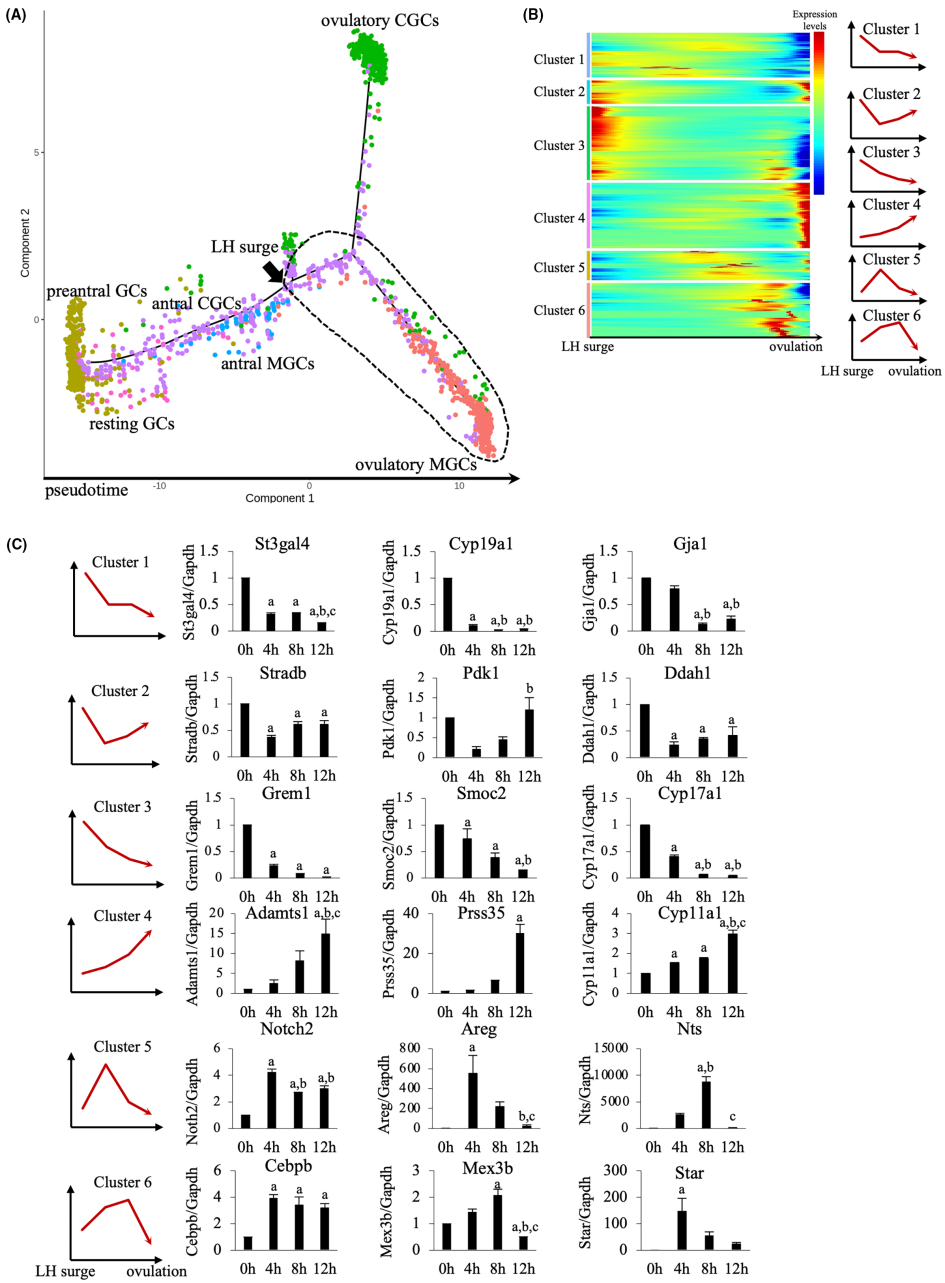
Differentiation of granulosa cell populations by pseudotime analysis. (A) Pseudotime analysis of single‐cell transcriptomes showing the differentiation process of granulosa cells from before the LH surge to after the LH surge. Cells were ordered along the differentiation process estimated by Monocle2 and colored the same color as the cell populations shown in Figure 1A. The time point of the LH surge (black arrow) was defined as the time when the dots of antral MGCs (blue dots) end. (B) Heatmap of gene expression during the differentiation of MGCs from the LH surge to ovulation (broken line circle in A). Genes with similar expression patterns were classified into six clusters. (C) mRNA expressions of representative genes in each cluster. Total RNA was isolated from MGCs obtained at 0, 4, 8, and 12 h after hCG injection in eCG‐hCG‐treated mice. We selected three representative genes in each cluster and measured their mRNA expressions by real‐time RT‐PCR. Values were normalized to those of *Gapdh* and expressed as a ratio of the 0 h sample. Data are mean ± SE of three mice. a, *p* < 0.05 versus 0 h; b, *p* < 0.05 versus 4 h; c, *p* < 0.05 versus 8 h.

We sorted six granulosa cell populations (resting GCs, preantral GCs, antral MGCs, antral CGCs, ovulatory MGCs, and ovulatory CGCs) during the differentiation from the LH surge to after the LH surge by pseudotime analysis (Figure 2A). The time point of the LH surge (black arrow in Figure 2A) was defined as the time when the dots of antral MGCs (blue dots in Figure 2A) end. As shown in Figure 2A, resting and preantral GCs differentiated into antral CGCs and antral MGCs before the LH surge, and after the LH surge, granulosa cells differentiated into two distinct cell populations, ovulatory MGCs and ovulatory CGCs. This suggests that ovulatory CGCs and MGCs acquire different functions after the LH surge.

To determine whether cell differentiation by pseudotime analysis reflects physiological cell differentiation, we decided to examine gene expression profiles during the differentiation of MGCs from the LH surge to ovulation (broken line circle in Figure 2A) because the changes in gene expression of MGCs during ovulation after the LH surge are well known. For this purpose, we classified the genes specifically expressed in this broken line circle into six clusters according to the pattern of gene expression changes (clusters 1–6 in Figure 2B). Then, three representative genes were selected in each cluster, and their mRNA expression levels were measured in granulosa cells obtained at 0, 4, 8, and 12 h after hCG injection in eCG‐hCG‐treated mice. The changes in mRNA expression of the representative genes were similar to the pattern of gene expression changes in each cluster that were determined based on the pseudotime analysis (Figure 2C). This indicates that our clustering and pseudotime analyses appropriately identify cell population and reflect physiological cell differentiation after the LH surge.

### 3.3 Cell–cell interactions between mural granulosa cells and cumulus granulosa cells

To investigate the interactions between MGCs and CGCs after the LH surge, interactome analysis was performed. We compared cell–cell interactions before and after the LH surge and identified the interactions that were activated after the LH surge. Among the identified interactions, there were 25 interactions in which ligands and receptors are expressed in both granulosa cells (Table 1). These were considered the bidirectional interactions. They were associated with the EGF receptor (EGFR) pathway, extracellular matrix, cell adhesion, cell differentiation, steroidogenesis, and cell morphology (Table 1). There were 26 interactions in which ligands are expressed in CGCs and receptors are expressed in MGCs (Table 2). These were considered as the interactions acting from CGCs toward MGCs. Because the expression levels of the representative ligands in MGCs were much lower than those in CGCs, these factors secreted from CGCs act as ligands in MGCs (Figure S1). They were associated with ovulation, steroidogenesis, extracellular matrix, cell adhesion, and inflammatory response (Table 2). There were 36 interactions in which ligands are expressed in MGCs and receptors are expressed in CGCs (Table 3). These were considered as the interactions acting from MGCs toward CGCs. They were associated with the EGFR pathway, hyaluronan synthesis, inhibition of steroidogenesis, extracellular matrix, cell adhesion, response to hypoxia, inflammatory response, oocyte maturation, and cell morphology (Table 3). These results suggest that MGCs and CGCs cooperatively regulate their functions in preovulatory follicles undergoing ovulation after the LH surge.

**TABLE 1:** Bidirectional interactions working between MGCs and CGCs.

Ligands from both GCs	Receptors on both GCs	Functions
Areg	Egfr	EGFR pathway
Btc	Egfr	
Epgn	Egfr	
Ereg	Egfr	
Fn1	ITGA5_ITGB1	Extracellular matrix formation
Lamb2	ITGA2_ITGB1	
Lamc1	ITGA2_ITGB1	
Lamc1	Dag1	
Col4a2	ITGA2_ITGB1	
Col6a1	ITGA2_ITGB1	
Col6a1	Sdc1	
Cdh2	Cdh2	Cell adhesion
Comp	Cd36	
Comp	Cd47	
Comp	Sdc1	
Comp	Sdc4	
Jam3	Jam3	
Angptl4	ITGA5_ITGB1	Cell differentiation
Angptl4	Sdc1	
Efna5	Epha5	
Efna5	Epha7	
Efnb2	Epha4	
Jag1	Notch2	
Retn	Cap1	Steroidogenesis
Cadm1	Cadm1	Cell morphology

**TABLE 2:** Interactions from CGCs to MGCs.

Ligands from CGCs	Receptors on MGCs	Functions
Bmp6	ACVR1_ACVR2B	Ovulation
Bmp6	ACVR1_BMPR2	
**Efna2**	**Epha4**	Steroidogenesis
**Efna2**	**Epha5**	
**Efna2**	**Epha7**	
**Efna4**	**Epha4**	
**Efna4**	**Epha5**	
**Efna4**	**Epha7**	
**Efna5**	**Epha4**	
**Wnt4**	**FZD1_LRP6**	
**Wnt4**	**FZD8_LRP6**	
Nampt	ITGA5_ITGB1	
Dll3	Notch2	
Lama1	ITGA2_ITGB1	Extracellular matrix formation
Fn1	Sdc1	
Agrn	Dag1	
Thbs1	Cd47	
Thbs1	Sdc1	
Thbs4	Cd47	
Thbs4	Sdc1	
Col4a2	Sdc1	
Jam2	Jam3	Cell adhesion
F11r	Jam3	
Sema5a	Plxna1	Inflammatory response
Sema7a	Plxnc1	
Spp1	ITGA5_ITGB1	

*Note*: The Bold terms are newly identified.

**TABLE 3:** Interactions from MGCs to CGCs.

Ligands from MGCs	Receptors on CGCs	Functions
Areg	EGFR_ERBB2	EGFR pathway
Btc	EGFR_ERBB2	
Ereg	EGFR_ERBB2	
**Tgfb1**	**ACVR1_TGFbR**	Hyaluronan synthesis
**Tgfb1**	**TGFbR1_R2**	
Gdf11	TGFBR1_ACVR2B	Inhibition of steroidogenesis
Tnfsf12	Tnfrsf12a	
Col4a1	ITGA2_ITGB1	Extracellular matrix formation
Col4a1	Sdc1	
Col4a1	Sdc4	
Col4a1	Cd44	
Col4a2	Sdc4	
Col4a2	Cd44	
Col6a1	ITGA9_ITGB1	
Col6a1	Sdc4	
Col6a1	Cd44	
Lama1	Cd44	
Lamb1	Cd44	
Lamb2	ITGA6_ITGB1	
Lamb2	Dag1	
Lamb2	Cd44	
Lamc1	ITGA6_ITGB1	
Lamc1	Cd44	
Fn1	ITGAV_ITGB1	
Fn1	Sdc4	
Fn1	Cd44	
Jam3	F11r	Cell adhesion
Jam3	Jam2	
Angptl4	Sdc2	Response to hypoxia
Angptl4	Sdc3	
Angptl4	Sdc4	
Sema3c	Plxnd1	Inflammatory response
Spp1	ITGA9_ITGB1	
Spp1	Cd44	
Jag1	Notch1	Oocyte maturation
Efnb2	Ephb4	Cell morphology

*Note*: The Bold terms are newly identified.

**FIGURE 3:**
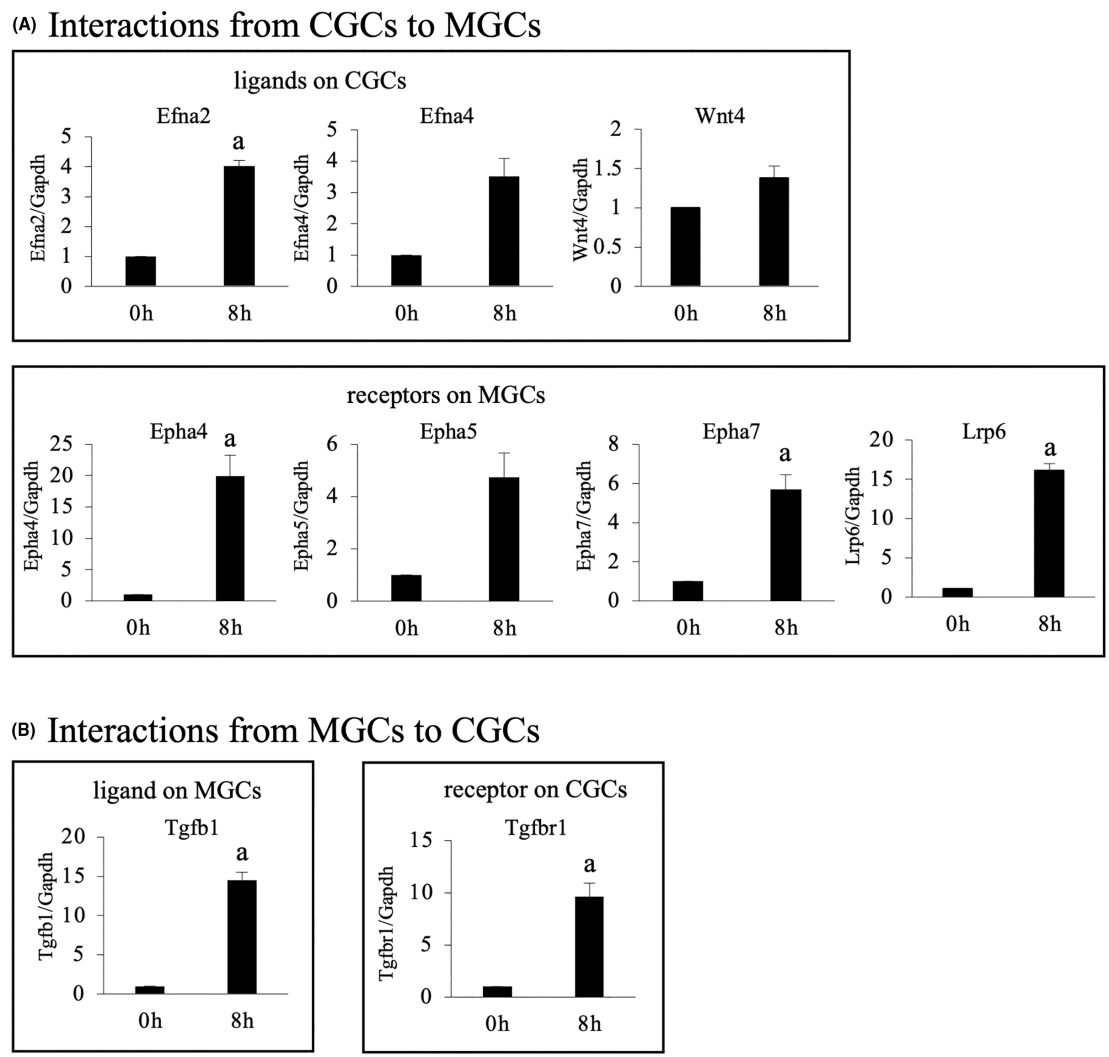
mRNA expressions of representative genes for the ligands and receptors in newly identified interactions. The ovaries were obtained at 0 and 8 h after hCG injection in eCG‐hCG‐treated mice. The follicles were punctured, and MGCs and COCs were collected, respectively. CGCs were stripped from oocytes by pipetting COCs in 0.1% Hyaluronidase. Total RNA was isolated from MGCs and CGCs. (A) Interactions acting from CGCs to MGCs. mRNA levels of *Efna2, Efna4*, and *Wnt4* as ligands on CGCs and *Epha4*, *Epha5, Epha7*, and *Lrp6* as receptors on MGCs were quantified by real‐time RT‐PCR. (B) Interactions acting from MGCs to CGCs. mRNA levels of *Tgfb1* as a ligand on MGCs and *Tgfbr1* as a receptor on CGCs were quantified by real‐time RT‐PCR. Values were normalized to those of *Gapdh* and expressed as a ratio of 0 h sample. Data are mean ± SE of three mice. a, *p* < 0.05 versus 0 h.

Interestingly, we found three novel interactions between MGCs and CGCs. Two are interactions of Efnas–Ephas and Wnt4‐FZDs_LRP6 from CGCs to MGCs, which are associated with steroidogenesis and lipid transport, respectively (bold type in Table 2). Another is an interaction of Tgfb1–TGFbRs from MGCs to CGCs, which is associated with hyaluronan synthesis (bold type in Table 3). Some of the genes for the ligands and receptors in the newly identified interactions were selected, and their mRNA expression levels were examined by real‐time RT‐PCR (Figure 3). They were significantly increased after the LH surge, suggesting that the interactions were activated after the LH surge.

## 4 DISCUSSION

The present study identified interactions between MGCs and CGCs in preovulatory follicles undergoing ovulation after the LH surge using in silico analysis of single‐cell RNA sequencing data. These interactions are involved in various processes including the EGF pathway, cell differentiation, steroidogenesis, cell morphology, extracellular matrix formation, cell adhesion, inflammatory response, and hyaluronan synthesis, and are likely to contribute to corpus luteum formation, oocyte maturation, and follicle rupture.^2^, ^3^, ^4^ In addition, we newly identified interactions between MGCs and CGCs, which were associated with steroidogenesis, lipid transport, and hyaluronan synthesis. Interestingly, our results showed that there are a number of interactions acting from CGCs toward MGCs, although accumulating data have shown the interaction acting from MGCs toward CGCs.

### 4.1 Identification of granulosa cell subtypes using single‐cell RNA sequencing analysis

Recently, single‐cell RNA‐seq technology has been used to clarify cellular diversity in various tissues. In this study, granulosa cells were clearly classified into not only different developmental stages of the ovary but also two distinct populations, MGCs and CGCs by clustering analysis using Seurat (Figure 1). In addition, pseudotime analysis using Monocle2 (1) estimated the process of cell differentiation, (2) allowed the granulosa cells to be divided before and after the LH surge, and (3) further divided into two branches, the ovulatory MGCs and the ovulatory CGCs (Figure 2). Our result by pseudotime analysis appears to be consistent with a report that CGCs and MGCs acquire different functions after the LH surge.^1^

### 4.2 Bidirectional interactions working between mural granulosa cells and cumulus granulosa cells

**FIGURE 4:**
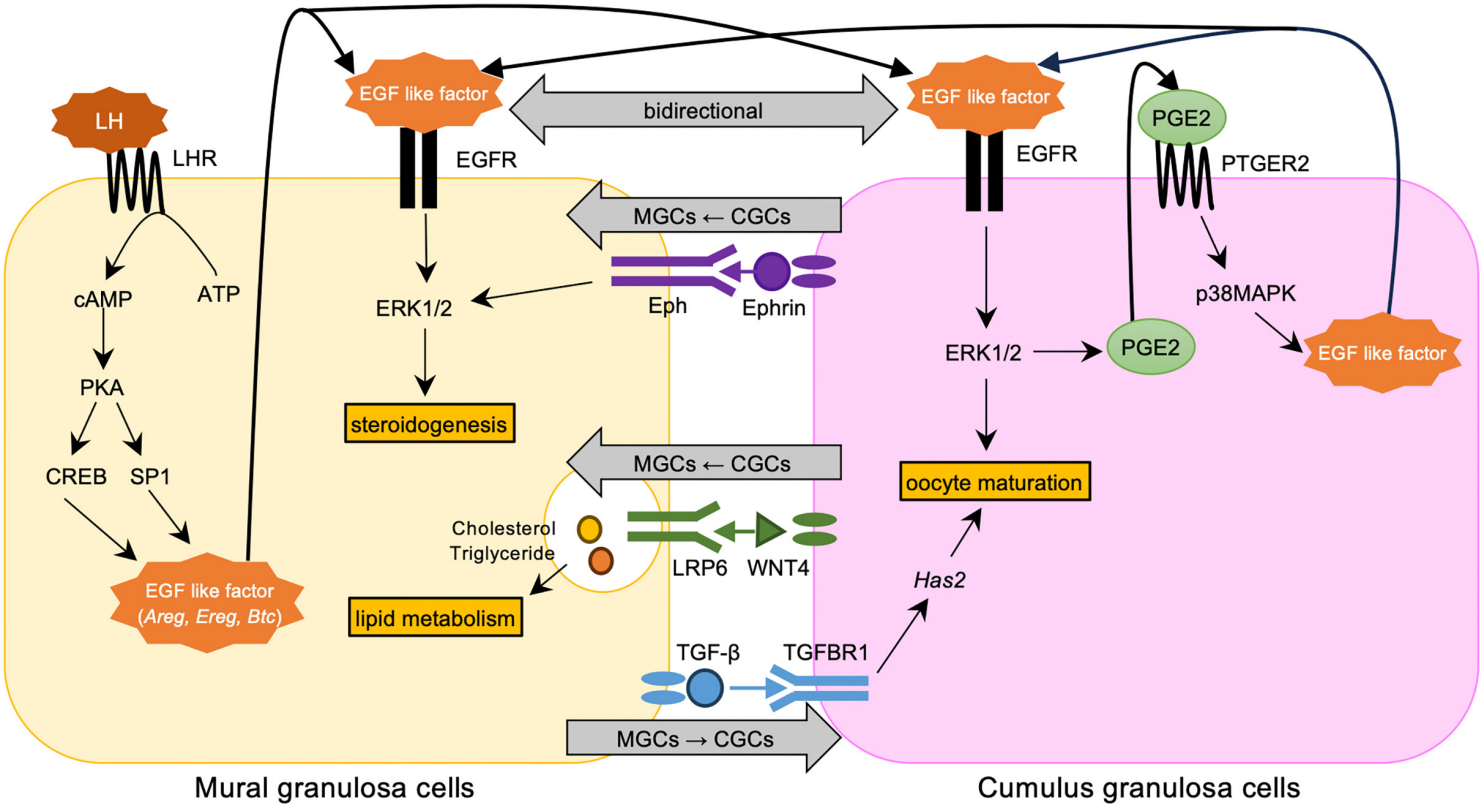
Schema showing interactions between MGCs and CGCs during ovulation after the LH surge. The EGFR pathway involves a bidirectional interaction between MGCs and CGCs. In brief, LH stimulates production of EGF‐like factors via cAMP‐PKA in MGCs, and then EGF‐like factors act on its receptor, EGFR, on both MGCs and CGCs, resulting in activation of the ERK1/2 pathway. Activated ERK‐1/2 is involved in steroidogenesis in MGCs, while it contributes to oocyte maturation and also stimulates production of EGF‐like factors through prostaglandin E2 (PGE2) in CGCs. EGF‐like factors produced by CGCs act on both MGCs and CGCs. Three novel interactions are shown. Ephrin–Eph pathway and Wnt–Lrp6 pathway from CGCs to MGCs, and TGF‐β–TGFBR1 pathway from MGCs to CGCs. Ephrin from CGCs binds to its receptor, Epha, on MGCs and is involved in steroidogenesis via ERK1/2 signaling in MGCs. WNT4 from CGCs acts on its receptor, LRP6 on MGCs and is involved in progesterone synthesis through lipid transport. TGF‐β1 from MGCs binds to TGFBR and induces hyaluronan synthesis by up‐regulation of Has2 expression in CGCs.

Our interactome analysis showed 25 bidirectional interactions in which ligands and receptors are expressed in both types of granulosa cells. One of the interactions involves the EGFR pathway, which is well known to be activated by the LH surge. EGF‐like factors and EGFR were expressed on both types of granulosa cells (Table 1). Figure 4 shows the schema of interactions between MGCs and CGCs during ovulation after the LH surge. LH stimulates production of EGF‐like factors via cAMP‐PKA in MGCs, and then EGF‐like factors act on its receptor, EGFR, on both MGCs and CGCs, resulting in activation of the ERK1/2 pathway.^5^, ^7^, ^32^, ^33^, ^34^, ^35^ Activated ERK‐1/2 is involved in steroidogenesis in MGCs,^36^ while it contributes to oocyte maturation^37^, ^38^ and also stimulates production of EGF‐like factors through prostaglandin E2 (PGE2) in CGCs.^6^, ^32^ EGF‐like factors produced by CGCs act on both MGCs and CGCs.

### 4.3 Interactions acting from cumulus granulosa cells toward mural granulosa cells

We identified 26 interactions in which ligands are expressed in CGCs and receptors are expressed in MGCs (Table 2). Among them, two interactions were novel. One of them involved the Efnas–Ephas pathway, which is associated with steroidogenesis (Table 2). Although EphrinA5 (*Efna5*) has been reported to be involved in the morphology and adhesion of mouse granulosa cells in vitro^39^ and corpus luteum formation in mice,^40^ it is not known if Efnas–Ephas has a role in the interactions between CGCs and MGCs. Several reports have indicated that the ERK‐1/2 pathway is activated by Efnas–Ephas signaling in other cell types.^41^, ^42^, ^43^ We speculate that Ephrins from CGCs bind to its receptor, Eph on MGCs and are involved in steroidogenesis via ERK1/2 signaling in MGCs after the LH surge (Figure 4).

Another novel interaction is the Wnt4‐FZDs_LRP6 pathway, which is associated with steroidogenesis (Table 2). LRP6 is a member of the low‐density lipoprotein receptor (LDLR) family and is also a receptor for Wnt/β‐catenin signaling.^44^ This signaling is called the canonical Wnt pathway. Previous reports showed that β‐catenin negatively regulates steroidogenesis during luteinization.^33^, ^45^ However, there is a β‐catenin‐independent pathway downstream of LRP6, called the non‐canonical pathway.^46^ Previous reports showed that non‐canonical pathway is involved in lipid metabolisms in mice liver.^47^ Therefore, we speculate that WNT4 from CGCs acts on its receptor, LRP6 on MGCs and activates the non‐canonical pathway to regulate lipid metabolisms in MGCs. Considering that cholesterol is a source of progesterone, the activated non‐canonical pathway may be involved in progesterone synthesis in MGCs after the LH surge (Figure 4). However, a previous report showed that WNT4 is also expressed in MGCs.^45^ Therefore, a further supply of WNT4 from CGCs may not have a significant role in MGCs. To clarify the role of WNT4 from CGC, future in vitro studies are needed to inhibit the activity of WNT4 secreted from CGC under the co‐culture of CGCs and MGCs.

It remains unknown whether CGCs act on MGCs in the preovulatory follicle undergoing ovulation after the LH surge, although it is well known that MGCs act on CGCs, for example, in oocyte maturation. Our results clearly showed that there are a number of interactions acting from CGCs toward MGCs. Further studies are needed to demonstrate whether the ligands on CGCs act on their receptors on MGCs, and what the role of their interactions is.

### 4.4 Interactions acting from mural granulosa cells toward cumulus granulosa cells

We identified 36 interactions in which ligands are expressed in MGCs and receptors are expressed in CGCs (Table 3). Among them, the Tgfb1–TGFbRs pathway was novel, which is associated with hyaluronan synthesis (Table 3). Expansion of the cumulus cell–oocyte complex (COC) in the preovulatory follicle requires induction of hyaluronan synthesis in CGCs.^48^ TGF‐β1 (*Tgfb1*) promotes hyaluronan synthesis by up‐regulating hyaluronan synthase 2 (*Has2*) expression in granulosa cells and cumulus cells in vitro.^49^, ^50^ However, it remains unclear whether TGF‐β1 synthesized by MGCs acts on CGCs as a paracrine factor. There is a possibility that TGF‐β1 from MGCs binds to TGFBR and induces hyaluronan synthesis by up‐regulation of *Has2* expression in CGCs, which contributes to COC expansion and the following oocyte maturation (Figure 4). Yang et al. previously reported that TGF‐β1 expression decreases in MGCs after the LH surge.^51^ This contributes to the oocyte maturation through the resumption of oocyte mitosis. However, they examined TGF‐β1 expression at 2 h after the LH surge, whereas we found the up‐regulation of TGF‐β1 at 8 h. Therefore, we speculate that TGF‐β1 from MGCs has biphasic effects on oocytes during luteinization, oocyte mitosis at the earlier phase, and COC expansion at the later phase.

In this study, using single‐cell RNA sequencing data, we identified cell–cell interactions between MGCs and CGCs, which induce key functional changes in preovulatory follicles after the LH surge leading to corpus luteum formation, oocyte maturation, and follicle rupture. Furthermore, our results strongly suggest that there are a number of interactions acting from CGCs toward MGCs in the preovulatory follicle undergoing ovulation after the LH surge, although the interaction acting from CGCs toward MGCs remains unknown so far.

## CONFLICT OF INTEREST STATEMENT

Norihiro Sugino is Editor‐in‐Chief of the Reproductive Medicine and Biology and co‐author of this article. He was excluded from the peer‐review process and all editorial decisions related to the acceptance and publication of this article. Peer review was handled independently by Editors‐in‐Associate Chief Tasuku Harada to minimize bias.

## ANIMAL STUDIES

This study was reviewed and approved by the committee for ethics on animal experiments at Yamaguchi University Graduate School of Medicine. All experiments were performed in accordance with relevant guidelines and regulations.

## Supporting information


Table S1.

Figure S1.
