# Supplementary figures and images for: Analysis of cell–cell interaction between mural granulosa cells and cumulus granulosa cells during ovulation using single‐cell RNA sequencing data of mouse ovary
Source: Reprod Med Biol. 2024 Feb 14;23(1):e12564. doi: 10.1002/rmb2.12564 (PMC10867398; doi:10.1002/rmb2.12564)

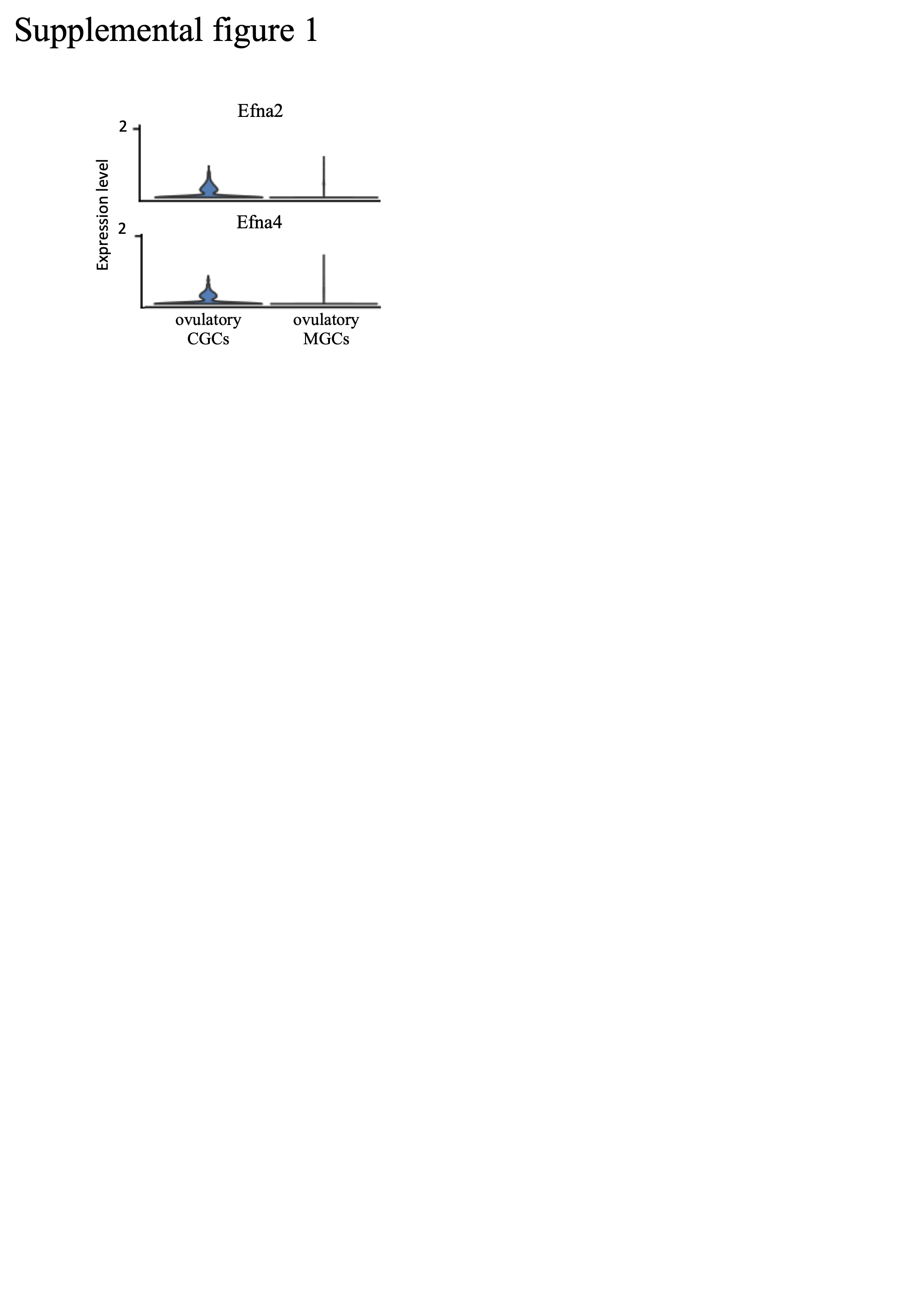

Supplement: Supplementary file 2 — Figure S1. [file RMB2-23-e12564-s002.tiff]
